# Isolation of Antimicrobial Compounds From *Cnestis ferruginea* Vahl ex. DC (Connaraceae) Leaves Through Bioassay-Guided Fractionation

**DOI:** 10.3389/fmicb.2019.00705

**Published:** 2019-04-11

**Authors:** Koffi Kouakou, Sujogya Kumar Panda, Ming-Rong Yang, Jing-Guang Lu, Zhi-Hong Jiang, Luc Van Puyvelde, Walter Luyten

**Affiliations:** ^1^UFR Biosciences, Université Félix Houphouët-Boigny, Abidjan, Côte d’Ivoire; ^2^Department of Biology, Katholieke Universiteit Leuven, Leuven, Belgium; ^3^State Key Laboratory of Quality Research in Chinese Medicine, Macau Institute for Applied Research in Medicine and Health, Macau University of Science and Technology, Macau, China

**Keywords:** *Cnestis ferruginea*, traditional african medicine, bioassay-guided fractionation, benzene-1,4-diol, caffeic acid methyl ester, *S. aureus*

## Abstract

Different parts of *Cnestis ferruginea* are used in traditional African medicine for treating infectious diseases such as dysentery, bronchitis, eye troubles, conjunctivitis, sinusitis, gonorrhea, and syphilis. Despite its long traditional use in the treatment of infections, this plant is not well studied for its *in vitro* antimicrobial properties. Therefore, the present study aims to establish the antimicrobial activity profile of extracts from this plant, as well as to isolate and evaluate the antimicrobial activity of the most abundant bioactive compound in *C. ferruginea* leaves through bioassay-guided purification, using *Staphylococcus aureus* as a target organism. Although both methanol and water extracts of the plant leaves proved active against *S. aureus*, a water extract was pursued, and subjected further to liquid-liquid partitioning (ethyl acetate, butanol, and water). The ethyl acetate fraction was found to be the most potent and was subjected to silica gel chromatography. In total, 250 fractions were obtained, and those with similar TLC profiles were clustered into 22 major groups, of which pooled fraction-F6 (83 mg) was the most potent. Additional purification by HPLC resulted in two active peaks, which were identified, using a combination of NMR and mass spectrometry, as hydroquinone and caffeic acid methyl ester. Their antimicrobial activity was confirmed using a microdilution protocol on *S. aureus*, where hydroquinone had a stronger activity (MIC_50_ = 63 μg/mL) compared to caffeic acid methyl ester (>200 μg/mL). Traditionally this plant is used as an aqueous preparation to treat many infections, and the present study also demonstrated antimicrobial activity in the aqueous extract, which appears due mainly to two major water-soluble compounds isolated through bioassay-guided purification. This supports the clinical use of the aqueous extract of *C. ferruginea* leaves as a phytotherapeutic for bacterial infections.

## Introduction

Despite advances in the discovery of natural and synthetic drugs, infectious diseases are still the leading cause of morbidity and death, especially in developing countries (Vargas et al., 2003). There is a progressive increase in strains of clinically important pathogens showing antibiotic resistance (Djeussi et al., 2013). Actions must be taken to reduce antimicrobial resistance and develop new antibiotics. There is renewed interest in natural products for the discovery of new bioactive substances with better pharmacological (including antibacterial) activities. These drug candidates may help to replace synthetic drugs with strong adverse effects (Adisa et al., 2011). On the other hand, sociocultural and economic reasons make traditional medicine the first choice for medication in developing countries. Scientists have developed new strategies to assess the pharmacological activity of plants, and they realize that starting form ethnopharmacologically used plants makes research more efficient and less expensive^1^. It is now understood by bioprospectors that tribal people represent a key to finding new and useful plants. The degree to which these indigenous peoples understand and can use the biodiversity that surrounds them (and in a sustainable manner) is remarkable. The essential components of medicine in Africa are plants (Mahomoodally, 2013).

*Cnestis ferruginea* (synonym C. *oblongifolia*) is an ornamental plant widely used under different local names, such as: “*wonsien blakassa*” by the Baule people of Côte d’Ivoire, “otito” by the Hausa tribe of Northern Nigeria, “amunkita” by the Igbo tribe of Southeast Nigeria, “Akara oje” and “Bonyin bonyin” by the Yoruba tribe of Southwest Nigeria (Yakubu and Ibiyo, 2013). “The bark yields a red dye which is used for dyeing clothing and the stems are used to make bows.”^2^ Different parts of the plant are used across tropical Africa to manage diverse ailments such as constipation (leaf or root), dysentery and gonorrhea (leaf), pains and inflammation (powdered bark), headache (root bark paste), bronchitis (leaf decoction), eye troubles (leaf sap), dysmenorrhoea (leaf or root), migraine and sinusitis (root sap or powder), toothache (root), conjunctivitis (fruit juice), abortion, sexual dysfunction and ovarian troubles (root decoction), rheumatism, stroke, and syphilis (Ahmed, 2017). The plant is well known as an emmenagog, abortifacient and aphrodisiac (N’guessan et al., 2006). The extract of the fruit is used to treat sore throat as a tonic, by mixing crushed fruits with rum or palm wine (a sweet fermented alcoholic sap obtained from palm trees used as a common beverage), as well as applied topically for the treatment of snakebite in Nigeria and Ghana. In Ivory Coast, the roots and leaves are employed in the treatment of dysmenorrhea, whereas in Nigeria the leaves are employed as a laxative. Pharmacological studies have shown that *C. ferruginea* possesses antibacterial (Boakye–Yiadom and Konning, 1975; Parvez and Rahman, 1992; Akharaiyi et al., 2012), anti-convulsant (Declume et al., 1984), antioxidant (Akharaiyi et al., 2012), anti-stress (Ishola and Ashorobi, 2007), analgesic and anti-inflammatory (Ishola et al., 2011) as well as hypoglycemic (Adisa et al., 2010) properties.

Despite its long traditional use in the treatment of infections, this plant is not well studied for the components that are responsible for its antimicrobial properties. Parvez and Rahman (1992) isolated an isoflavone glycoside: afrormosin-7-O-beta-D-galactoside, which possesses broad antimicrobial activity, though only at concentrations above 1 mg/mL. Since then, no more study was undertaken to find out the antimicrobial compounds. Hence, the objective of the present study was to isolate the main antimicrobial constituents from *Cnestis ferruginea* leaves through bioassay-guided purification.

## Materials and Methods

### Standard and Reagents

Solvents (hexane, EtOAc, acetone, butanol, methanol, acetonitrile) were of HPLC grade purity and were from Sigma-Aldrich Co. (United States). Sterile deionised water was produced by a water purification system (Milli-Q Reagent Water System, MA, United States). Mueller–Hinton (MH) broth was purchased from Lab M Ltd. (Lancashire, United Kingdom). DMSO (molecular biology grade), hydroquinone, and ciprofloxacin hydrochloride were purchased from Sigma-Aldrich (MO, United States).

### Collection and Identification of Plants

**FIGURE 1:**
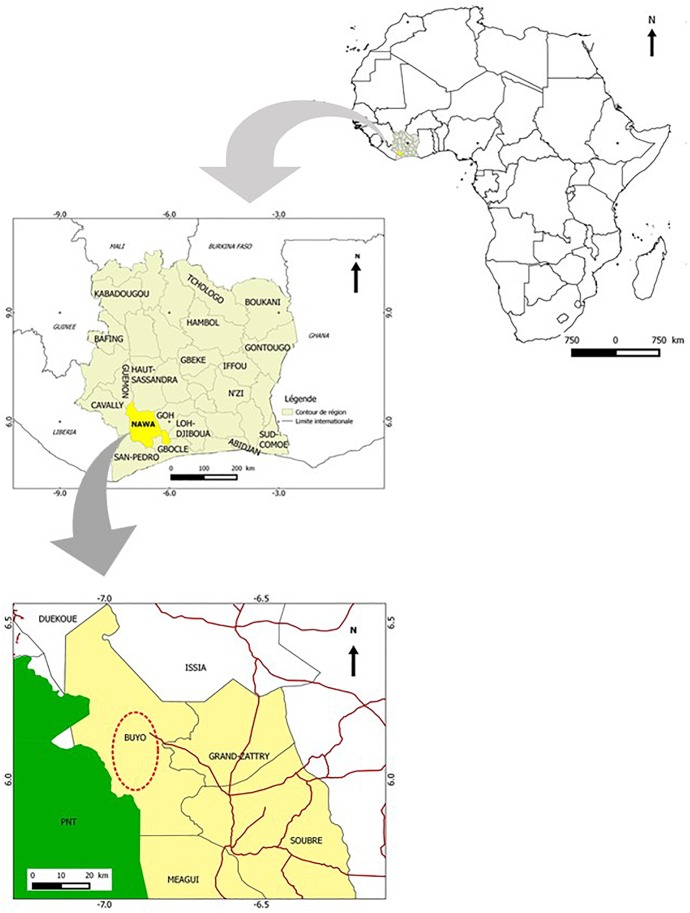
Maps showing the area of collection of *Cnestis ferruginea.*

Fresh leaves of *Cnestis ferruginea* Vahl ex. DC (Connaraceae) were collected during September, 2014 from Buyo, Nawa, Ivory Coast (Figure 1). The plant material was identified using descriptive references of the flora of Côte d’Ivoire (Aké Assi, 2001) and a voucher specimen (CSRS001969) was deposited at the Centre Suisse de Recherche Scientifique, *Côte d’Ivoire.* The leaves were dried at ambient temperature to maintain their green color and volatile oils (Panda, 2014).

### Small-Scale Extraction

The dried raw botanical material was ground to a fine powder. Small-scale extractions were performed as described in a previous study (Panda et al., 2017). Briefly, one gram of powder was transferred separately into each of four 15 mL sterile Falcon tubes, and extracted with four different solvents: acetone, hexane, ethanol, and water. One mL aliquots or each extract were dried by evaporation of the solvent (Savant SpeedVac Concentrator 200H, Stratech Scientific, London, United Kingdom), and the dried residue was re-dissolved in 200 μL DMSO for the organic solvent extracts, and in 200 μL water for the aqueous extract. All the samples were stored at 4°C until further testing (Panda et al., 2017).

### Culture and Antibacterial Activity

The bacterial strains used in this study were: *Aeromonas hydrophila* ATCC 7966, *Bacillus cereus* LMG 9610, *Citrobacter freundii* ATCC 8090, *Klebsiella pneumoniae* ATCC 13883, *Micrococcus lysodeikticus* ATCC 4689, *Pseudomonas aeruginosa* LMG 14083, *Pseudomonas aeruginosa* PA01, *Shigella flexneri* LMG 10472, *Staphylococcus aureus* ATCC 12600, *Staphylococcus aureus* ATCC 6538, and *Staphylococcus epidermidis* LMG 10273. Antibacterial activity was assessed as described in a previous study (Panda et al., 2017) using a microdilution broth protocol.

### Large-Scale Extraction (Successive)

Three hundred and fifty grams of the dried plant was weighed and transferred to a 2.5 L glass bottle; 1 L hexane was added and thoroughly mixed with the plant material. The bottle was placed in a sonication water bath for four times 30 min every 6 h to improve extraction. Then, the extract was filtered (185 mm, MACHEREY-NAGEL, Germany) and evaporated on a rotavapor. This was repeated until the extract yield became negligible. After hexane extraction, the residual plant material was extracted in the same manner with a second solvent (acetone), followed by extractions with methanol and water. The water extract was, in turn, again mixed with an equal volume of saturated EtOAc, and shaken vigorously in a separatory funnel, followed by spontaneous phase separation and recovery of the lower phase. This step was repeated three times, resulting in the separation of the EtOAc and water phase. Subsequently, the water phase was extracted in a similar way with saturated butanol in a liquid-liquid extraction (Supplementary Figure S1). All these fractions were concentrated under reduced pressure using a BUCHI rotavapor R-100.

### Bioassay-Guided Purification

The dried active extract (EtOAc) was adsorbed on silica gel (70 to 230 mesh) and loaded on a silica gel column (600 mm height × 55 mm diameter), which was eluted (Waters, model 600) with a step gradient increasing in polarity- hexane: dichloromethane, 9.5:0.5, 9:1, 8.5:0.5, 8:2, 7:3; 6:4, 5:5, 4:6, 3:7, 2:8, 1:9, and 0:10. The column was then eluted with 100% dichloromethane, then 100% EtOAc, followed by a mixture of EtOAc and methanol (9.5:0.5, 9:1, 8:2, 7:3, 6:4, 5:5, 4:6, 3:7, 2:8, and 1:9). Finally, the column was eluted with 100% methanol (Supplementary Figure S1). In each step, ten tubes of 40 mL fractions were collected, and the solvent was evaporated. The whole separation was monitored by a Dual λ model 2487 absorbance detector at 280 nm and 254 nm.

### Thin Layer Chromatography

Aliquots (5 μL) of the 245 fractions from the EtOAc extract were spotted on large TLC glass plates (Sigma Aldrich, Germany, dimensions L × W 20 × 20 cm). The spotted plates were developed in glass jars (20 × 10 × 20 cm), pre-saturated with the chosen mobile phase at ambient temperature, and dried in an oven at 90°C for 5 min to remove the solvent. The plates were visualized under ultra-violet (UV) light at 254 and 360 nm, followed by spraying with 5% sulfuric acid in ethanol, followed by heating at 100°C for 5 min. Fractions with similar TLC patterns were pooled for biological activity testing. In total, 22 pooled fractions were prepared and further tested for antimicrobial activity against *S. aureus* at a concentration of 1000 μg/mL.

### Antibacterial Activity and Determination of Minimum Inhibitory Concentration (MIC_50_)

Dried extracts, dissolved in DMSO, were stored at 4°C and brought to ambient temperature right before the start of each experiment. After thorough vortexing of the tubes with stored extracts, a two–fold serial dilution up to 32–fold was prepared in a 96–well conical bottom (V) microtiter plate using DMSO, and activity testing was performed in 96-well flat-bottom microtiter plates (Costar, made in United States). Data from dose-response experiments were represented as the percentage of inhibition compared to the solvent control, and analyzed with Prism^TM^ (GraphPad Prism 5.0 Software Inc., San Diego, CA, United States). The IC_50_ for each growth condition was calculated by fitting the data to a non-linear least-squares sigmoid regression curve (Panda et al., 2018), fixing the top and bottom of the curve at 100 and 0 percent, respectively. The MIC_50_ corresponds to the concentration that would yield an inhibition of 50%.

### HPLC-DAD Analysis

High performance liquid chromatography analyses were performed on a Shimadzu, LC-20AT system (model DGU 20A3, Japan) equipped with LC-20AT quaternary pump, a DGU-20A3/DGU-20A5 on-line degasser, a SPD-20A photodiode array detector, and a CBM-20A/20A interface. The data were acquired and processed using Lab Solution software. The active fraction was analyzed using a reverse-phase HPLC column: Sunfire^TM^ prep C18 (10 mm × 250 mm, 5 μm) column (Waters, Made in Ireland). The mobile phase was composed of 30% H_2_O with 0.1% TFA (ACROS ORGANICS) and 70%acetonitrile (LC-MS *CHROMASOLV*^®^, Fluka) with 0.1% TFA. Prior to HPLC injection, samples were filtered through a CHROMAFIL^®^ Xtra H-PTFI filter (pore size 0.45 μm, filter 13 mm, MACHEREY-NAGEL, Germany). Two mL of sample was injected and run for 41 min at 20°C with flow rate of 4 mL/min (Kerkoub et al., 2018).

### The Ultra-High Performance Liquid Chromatography-Quadrupole Time-of-Flight Mass Spectrometry (UHPLC-QTOF MS) Analysis

Liquid chromatography mass spectrometry analyses were performed on an Agilent 1290 Infinity UHPLC system (Agilent Technologies, Santa Clara, CA, United States) coupled with a Bruker Maxis Impact Q-TOF Mass Spectrometer (Bruker, Switzerland). The chromatographic separation was performed on an Agilent poroshell 120 EC-C_18_ column (150 mm × 3.0 mm, 2.7 μm). The mobile phase consisted of 0.1% formic acid in water (A) and 0.1% formic acid in acetonitrile (B). A linear gradient was optimized as follows: 0–8 min, 5–95% B; 8–10 min, 95% B; 10–11 min, 95–5% B, and finally equilibration with 5% B for 3 min at a flow rate of 0.35 mL/min. The injection volume was 5 μL with a concentration of 1 μg/mL, and the column temperature was maintained at 30°C. QTOF-MS conditions: ESI mass spectra were acquired in positive and negative mode. The ESI source parameters were set as follows: end plate offset voltage of 500 V, capillary voltage of 4000 V, nebulizer of 2.5 bar and dry gas flow of 8.0 L/min 1 at 200°C (Kerkoub et al., 2018).

### NMR Spectroscopy

^1^H NMR (600 MHz) and ^13^C NMR (150 MHz) spectra were acquired with a Bruker Ascend LH 600 MHz NMR spectrometer (Bruker, Switzerland) equipped with a 5 mm CryoProbe (CP DCH 600S3 C/H-D-05 Z) in deuterated methanol solution at 298°K. Chemical shifts are given in δ (ppm), and coupling constants (*J*) are expressed in Hertz (Hz) (Kerkoub et al., 2018).

## Results

Four different extracts viz. acetone, hexane, ethanol and water, were initially prepared in parallel from the leaves of *C. ferruginea* and tested for antibacterial activity (*in vitro)* against a panel of pathogenic bacteria (Table 1). The water extract had the broadest-spectrum activity, inhibiting all test pathogens except *S. epidermidis*, followed by the acetone and ethanol extracts. The hexane extract was only active against *Klebsiella pneumoniae*. Therefore, a large-scale preparation was made by successive extraction using hexane, followed by acetone, methanol, and water. The (active) water fraction was further partitioned with EtOAc and butanol (see Supplementary Figure S1).

**Table 1:** Antibacterial activity of small-scale extracts of *C. ferruginea.*

Strain information	Acetone	Ethanol	Hexane	Water
*Aeromonas hydrophila* ATCC 7966	+	−	−	+
*Bacillus cereus* LMG 9610	+	+	−	+
*Citrobacter freundii* ATCC 8090	+	−	−	+
*Klebsiella pneumoniae* ATCC 13883	+	+	+	+
*Micrococcus lysodeikticus* ATCC 4689	−	−	−	+
*Pseudomonas aeruginosa* LMG 14083	+	+	−	+
*Pseudomonas aeruginosa* PA 01	+	+	−	+
*Shigella flexneri* LMG 10472	+	−	−	+
*Staphylococcus aureus* ATCC 12600	−	−	−	+
*Staphylococcus aureus* ATCC 6538	−	+	−	+
*Staphylococcus epidermidis* LMG 10273	−	+	−	−

*( + ) antimicrobial activity; (−) no activity.*

**FIGURE 2:**
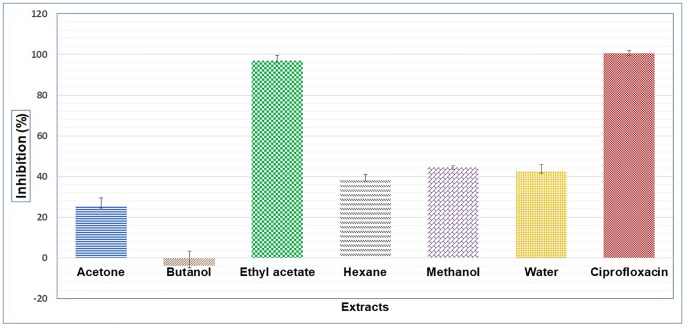
Antibacterial activity on *S. aureus* of different extracts prepared with liquid-liquid separation of an aqueous extracts of *C. ferruginea* leaf; positive control ciprofloxacin.

**FIGURE 3:**
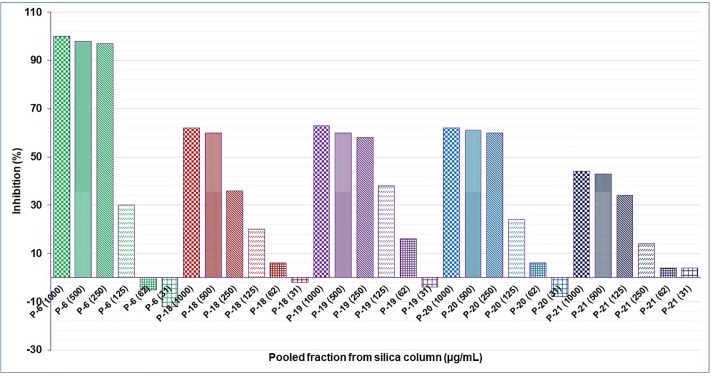
Anti-staphylococcal activity of serial dilution of the active pooled fractions from a silica gel column of the EtOAc subfraction of an aqueous extract of *C. ferruginea.*

**FIGURE 4:**
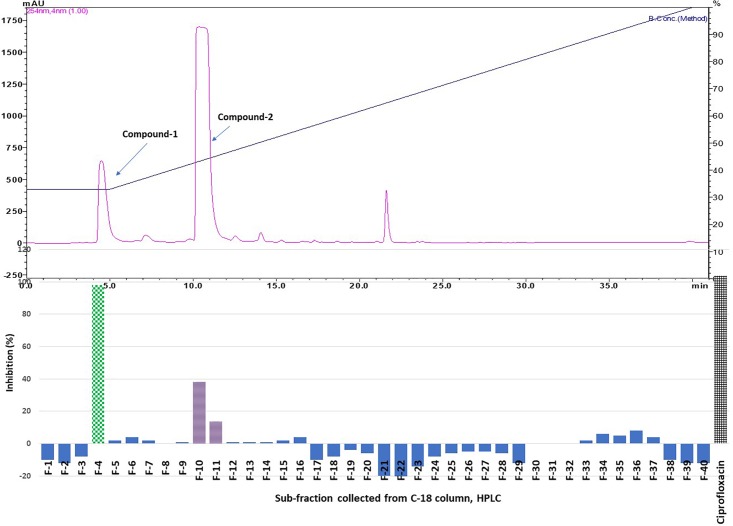
Top panel: HPLC chromatogram of pooled fraction 6 (P 6) of silica gel column; fractions were collected per minute and tested for activity (percentage inhibition of *S. aureus*) (bottom panel); positive control ciprofloxacin.

Results showed that of the six partition fractions of the large-scale extract, the EtOAc fraction was the most potent (100% inhibition against *S. aureus*), while moderate inhibition was observed with the hexane, methanol, and water partition fractions (Figure 2). Therefore, the EtOAc partition fraction was further separated into 300 fractions by silica gel chromatography. Fractions between 48 and 300 with similar TLC profiles were grouped into 22 major pools. When tested against *S. aureus*, pools (P) 6, 18, 19, 20 and 21 were found to be active (Table 2). These pools were dried and weighed: P6 (83.7 mg), P18 (247.9 mg), P19 (110 mg), P20 (40 mg), and P21 (4 mg). An MIC_50_ was determined for all these pooled fractions using a serial dilution protocol, starting with a concentration of 1000 μg/mL (Table 2). Pool 6 was the most active with an MIC_50_ of 158 μg/mL (Table 2 and Figure 3). Therefore, F6 was further separated using analytical HPLC (see section 2.8) with a water:acetonitrile mobile phase (starting at 30% acetonitrile for 5 min and linearly increasing to 100% over 40 min). The chromatogram recorded at 254 nm showed three well-resolved peaks (Figure 4). All 41 one-minute fractions were collected and re-tested against *S. aureus*, yielding two fractions with antibacterial activity (Figure 4).

**Table 2:** MIC_50_ against *S. aureus* of pooled fractions from silica gel column.

Name of the pool fraction	Antibacterial activity (% inhibition) at 1000 μg/mL	MIC_50_ (μg/mL)
Pool fraction 1 (P1)	−10	–
Pool fraction 2 (P2)	−12	–
Pool fraction 3 (P3)	−14	–
Pool fraction 4 (P4)	−6	–
Pool fraction 5 (P5)	−2	–
Pool fraction 6 (P6)	100	158
Pool fraction 7 (P7)	−21	–
Pool fraction 8 (P8)	−5	–
Pool fraction 9 (P9)	−8	–
Pool fraction 10 (P10)	2	–
Pool fraction 11 (P11)	−6	–
Pool fraction 12 (P12)	−5	–
Pool fraction 13 (P13)	−8	–
Pool fraction 14 (P14)	0	–
Pool fraction 15 (P15)	−4	–
Pool fraction 16 (P16)	−6	–
Pool fraction 17 (P17)	−8	–
Pool fraction 18 (P18)	62	443
Pool fraction 19 (P19)	63	175
Pool fraction 20 (P20)	62	272
Pool fraction 21 (P21)	44	316
Pool fraction 22 (P22)	−10	–

**FIGURE 5:**
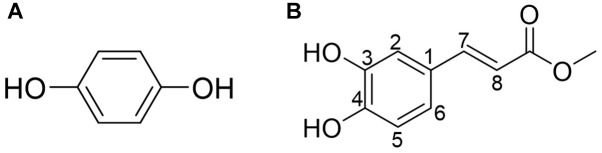
**(A)** Compound-1 (hydroquinone). **(B)** Compound-2 (caffeic acid methyl ester). Compounds 1 and 2 in the most active peaks were analyzed by mass spectrometry and NMR (see Supplementary Material).

Purified active peaks were subjected to NMR and LC-MS for identification. Compound-1 (hydroquinone): ^1^H-NMR (600 MHz, CD_3_OD, ppm) δ: 6.62 (4H, s, H-2, 3, 5, 6). ^13^C-NMR (150 MHz, CD_3_OD, ppm) δ: 116.8 (C-2, 3, 5, 6), 151.2 (C-1, 4) (Figure 5A). Compound-2 (caffeic acid methyl ester): positive-ion HRESIMS *m/z* 195.0655 [M + H]^+^ (calcd; 195.0652 for C_10_H_10_O_4_), negative-ion HRESIMS *m/z* 193.0502 [M-H]^-^ (calcd; 193.0506 for C_10_H_10_O_4_). ^1^H-NMR (600 MHz, CD_3_OD, ppm) δ: 3.75 (3H, s, OCH_3_ ), 6.27 (1H, d, *J* = 15.8Hz, H-7), 6.78 (1H, d, *J* = 8.2Hz, H-5), 6.94 (1H, dd, *J* = 2.0, 8.2Hz, H-6), 7.03 (1H, d, *J* = 2.0Hz,H-2), 7.55 (1H, d, *J* = 15.8Hz, H-8). ^13^C-NMR (150 MHz, CD_3_OD, ppm) δ: 52.0 (OCH_3_), 114.8 (C7), 115.1 (C2), 116.5 (C5), 122.9 (C6), 127.7(C1), 146.8 (C3), 146.9 (C8), 149.6(C4), 169.8 (CO) (Figure 5B). The antimicrobial properties of both compounds were quantified using a microdilution test on *S. aureus*. Hydroquinone had a stronger activity MIC_50_ = 63 μg/mL) compared to caffeic acid methyl ester (MIC_50_ > 200 μg/mL).

## Discussion

*Cnestis ferruginea* is well known for its antibacterial property, and has been used traditionally to treat several infectious diseases. According to Boakye–Yiadom and Konning (1975), the root, stem, and leaves of this plant have broad-spectrum activity against *B. subtilis, S. aureus, E. coli*, and *P. aeruginosa*. The leaf extracts (especially in water or acetone) also showed activity against a broad range of Gram-positive as well as Gram-negative human pathogens. Extracts of *C. ferruginea* showed strong antifungal activity against *Aspergillus niger* (Le Grand et al., 1988). Ndukwe et al. (2005) reported activity in the fruits of *C. ferruginea* against multiple bacteria responsible for orofacial infection. Although the antimicrobial activity of *C. ferruginea* has been well documented, no one thus far has identified bioactive compounds responsible for this antimicrobial activity, except for Parvez and Rahman (1992). However, the compound they purified had weak activity (see below). Ishola et al. (2011) used bioactivity-guided purification from root extract to identify the compound responsible for analgesic and anti-inflammatory activity as amentoflavone. The present study was designed to investigate the antibacterial compounds from the leaves of *C. ferruginea* using bioassay-guided purification.

The phytochemistry of *C. ferruginea* has not been studied well so far. In 1982, Olugbade et al. (1982) first reported the presence of coumarin, flavonoids, squalene, β-sitosterol and triacontanol in the roots. Later, Ogbede et al. (1986) and Ogbechie et al. (1987), isolated a few additional phytocompounds such as apigenidin, cyanidin, delphinidin, octacosanyl stearate, myricyl alcohol and 1-myristo-2-stearo-3-palmitin. Subsequently, Parvez and Rahman (1992) isolated from the fruit extract a novel isoflavone glycoside: afrormosin-7-O-β-D-galactoside, which demonstrated antimicrobial activity against *S. aureus*, *E. coli* and *Candida albicans*, but only at high concentrations (inhibition ranging from 40 to 75% for concentrations ranging from 1 to 5 mg/mL).

Using bioassay guided fractionation, the present study led to the isolation of two antimicrobial compounds: benzene-1,4-diol (compound 1) and caffeic acid methyl ester (compound 2), whose MIC_50_ is 63 and > 200 μg/mL, respectively. Although these MIC_50_ values are relatively high, these two compounds are the major contributors to the antibacterial effect. The antibacterial isoflavone galactoside isolated by Parvez and Rahman showed considerably lower potency for *S. aureus* (45 and 60% inhibition at 1000 and 3000 μg/mL, respectively). Since these authors did not report a yield, it is not possible to determine the contribution of this compound to the overall antimicrobial effect. Moreover, their compound was isolated from fruits, and it is not clear whether it is even present in leaf extracts. In any case, this appears to be the first demonstration of caffeic acid methyl ester in (leaves of) *C. ferruginea*. Adisa and Olorunsogo (2013) isolated hydroquinone from the leaves of *C. ferruginea* but did not study its antimicrobial (but rather its antioxidant) effects. This study is the first report on the isolation from *C. ferruginea* of hydroquinone, responsible for the antibacterial activity, using bioassay-guided purification.

In select edible Korean plant seeds, Kim et al. (2010), found antibacterial activity of several quinone derivative including hydroquinone, with an MIC of 63 μg/mL against *S. aureus*, similar to the present study. In the same year, Rúa et al. (2010) also reported that “hydroquinone was the strongest inhibitor of the 19 phenolic compounds that were tested against *S. aureus*.” A year later, the anti-staphylococcal activity of hydroquinone was tested against several strains of *S. aureus* obtained from ATCC, and observed to range from 62.5 to 100 μg/mL (Rúa et al., 2011). Rúa and his co-workers concluded that hydroquinone has greater potency than other antimicrobial phenolics like thymol, carvacrol, butylated hydroxyanisole, and gallic acid (Rúa et al., 2010), some of which are used clinically (thymol) or as preservative in food (carvacrol).

Jeyanthi et al. (2016), isolated hydroquinone (benzene-1,4-diol) from halotolerant *Bacillus methylotrophicus* MHC10 and found strong inhibitory activity against both Gram-positive and Gram-negative bacteria. The cytotoxicity of hydroquinone appears to occur through an oxidative mechanism based on its ability to undergo autoxidation, leading to quinone formation with the production of reactive oxygen species (Urios et al., 2003). Recently, Jurica et al. (2017), studied the antimicrobial property of strawberry trees and concluded that arbutin did not show direct antimicrobial activity, while its metabolite hydroquinone showed strong antimicrobial activity against *Enterococcus faecalis.*

Hydroquinone is produced industrially on a large scale, and is used extensively as a reducing agent, as a photographic developer, as a stabilizer or polymerizing inhibitor for certain materials, as antioxidant, and as skin-lightening agent in cosmetics, hair dyes, and medical preparations. It degrades easily both by photochemical and biological processes, and as a result does not persist in the environment (World Health Organization, 1996). High-level exposure to hydroquinone causes severe effects on the central nervous system and possibly death (DeCaprio, 1999). Acute toxicity was found with oral LD_50_ values of 245 mg/kg in the mouse, 320 mg/kg in the rat, 200 mg/kg in the dog, 70 mg/kg in the cat, and 550 mg/kg in the guinea pig (Devillers et al., 1990; DeCaprio, 1999) while in the Sprague-Dawley rat the LD50 was >375 mg/kg (Topping et al., 2007). Many years ago, a controlled oral study on human volunteers, conducted by Carlson and Brewer (1953), observed that ingestion of 300–500 mg hydroquinone daily for 3–5 months did not produce any observable pathological changes in blood or urine. The compound therefore appears relatively safe.

Using bioassay-guided fractionation, the second-most active antimicrobial compound isolated from *C. ferruginea* leaf is caffeic acid methyl ester. It is an ester of a naturally occurring phenolic compound and has previously been isolated from plants such as *Echinops hispidus* (Muithya, 2010)*, Lavatera trimestris* and *Leucaena leucocephala* (Ting et al., 2014), but (to the best of our knowledge) not from *C. ferruginea*. Although the anti-staphylococcal activities of caffeic acid derivatives (including its methyl ester) have already been reported (Rahman and Moon, 2007; Ting et al., 2014), their mechanism of action is not fully understood. Recently, Ge et al. (2018) isolated caffeic acid methyl ester from *Lonicera japonica* flowers and reported that it significantly reduced hepatitis B virus. Even though many derivatives of caffeic acid or its analogs are also biologically active, caffeic acid methyl ester is still an important compound due to its diverse biological activities (including cytotoxicity). Nonetheless, modifications of the caffeic acid structure into esters or amides can generate novel analogs with improved and more selective biological activity (Touaibia et al., 2011), particularly as antimicrobials (Fu et al., 2010). In the present study, caffeic acid methyl ester showed anti-staphylococcal activity, but only at fairly high concentrations. Even at 200 μg/mL (>1000 μM) we did not achieve full growth inhibition. However, during bioassay-guided fractionation it was picked up, presumably due to its abundant presence, and perhaps because it may have (partly) oxidized.

## Conclusion

In conclusion, aqueous extracts of *C. ferruginea* leaves showed antimicrobial activity due to the presence of hydroquinone and caffeic acid methyl ester. This supports its traditional use for infections and confirmed that the active molecules are water-soluble.

## Author Contributions

KK, SP, LVP, and WL conceived and designed the experiments. SP, KK, M-RY, and J-GL performed the experiments. SP, LVP, WL, M-RY, J-GL, and Z-HJ analyzed the data. WL and Z-HJ contributed reagents, materials, and analysis tools. KK, SP, WL, M-RY, Z-HJ, and LVP contributed to the writing of the manuscript. All authors contributed to manuscript revision, read, and approved the submitted version.

## Conflict of Interest Statement

The authors declare that the research was conducted in the absence of any commercial or financial relationships that could be construed as a potential conflict of interest.

## Footnotes

1 http://csc.columbusstate.edu/summers/Outreach/RainSticks/fRainforestFacts.htm

2 http://tropical.theferns.info/viewtropical.php?id=Cnestis+ferruginea

## Abbreviations

ATCC: American Type Culture Collection, Manassas, Virginia, United States
DMSO: dimethyl sulphoxide
ESI: electrospray ionization
EtOAc: ethyl acetate
HPLC: high performance liquid chromatography
LC-MS: liquid chromatography mass spectrometry
MHA: mueller–hinton agar
MIC: minimum inhibitory concentration
NMR: nuclear magnetic resonance
OD: optical density
TFA: trifluoroacetic acid
UHPLC-QTOF MS: ultra high performance liquid chromatography-quadrupole time-of-flight mass spectrometry.
