# Supplementary material for: Isolation of Antimicrobial Compounds From Cnestis ferruginea Vahl ex. DC (Connaraceae) Leaves Through Bioassay-Guided Fractionation
Source: Front Microbiol. 2019 Apr 11;10:705. doi: 10.3389/fmicb.2019.00705 (PMC6470257; doi:10.3389/fmicb.2019.00705)
Supplement: Supplementary file 1 [file Data_Sheet_1.docx]

Supplementary Material

# Isolation of antimicrobial compounds from *Cnestis ferruginea* Vahl ex. DC (Connaraceae) leaves through bioassay-guided fractionation

Koffi Kouakou^1¥*^, Sujogya Kumar Panda^2¥*^, Ming-Rong Yang^3^, Jing-Guang Lu^3^, Zhi-Hong Jiang^3^, Luc Van Puyvelde^2^, Walter Luyten^2^

^1^ UFR Biosciences, Université Félix Houphouët-Boigny (UFHB), Côte d’Ivoire

^2^ Department of Biology, KU Leuven, Leuven, Belgium

^3^ State Key Laboratory of Quality Research in Chinese Medicine, Macau Institute for Applied Research in Medicine and Health, Macau University of Science and Technology, Macau, China

*Correspondence

Dr. Koffi Kouakou

[kouakoukoff@yahoo.fr](mailto:kouakoukoff@yahoo.fr)

Dr. Sujogya Kumar Panda

[sujogyapanda@gmail.com](mailto:sujogyapanda@gmail.com)

¥ Authors contributed equally

**Supplementary materials:**

Figure 1: Extraction and separation of phytochemicals for bioassay guided fractionation

Figure 2: ^1^H-NMR spectrum of compound 1

Figure 3: ^13^C-NMR spectrum of compound 1

Figure 4: HRMS spectrum of compound 2

Figure 5: ^1^H-NMR spectrum of compound 2

Figure 6: ^13^C-NMR spectrum of compound 2

Figure 1: Extraction and separation of phytochemicals for bioassay guided fractionation

*
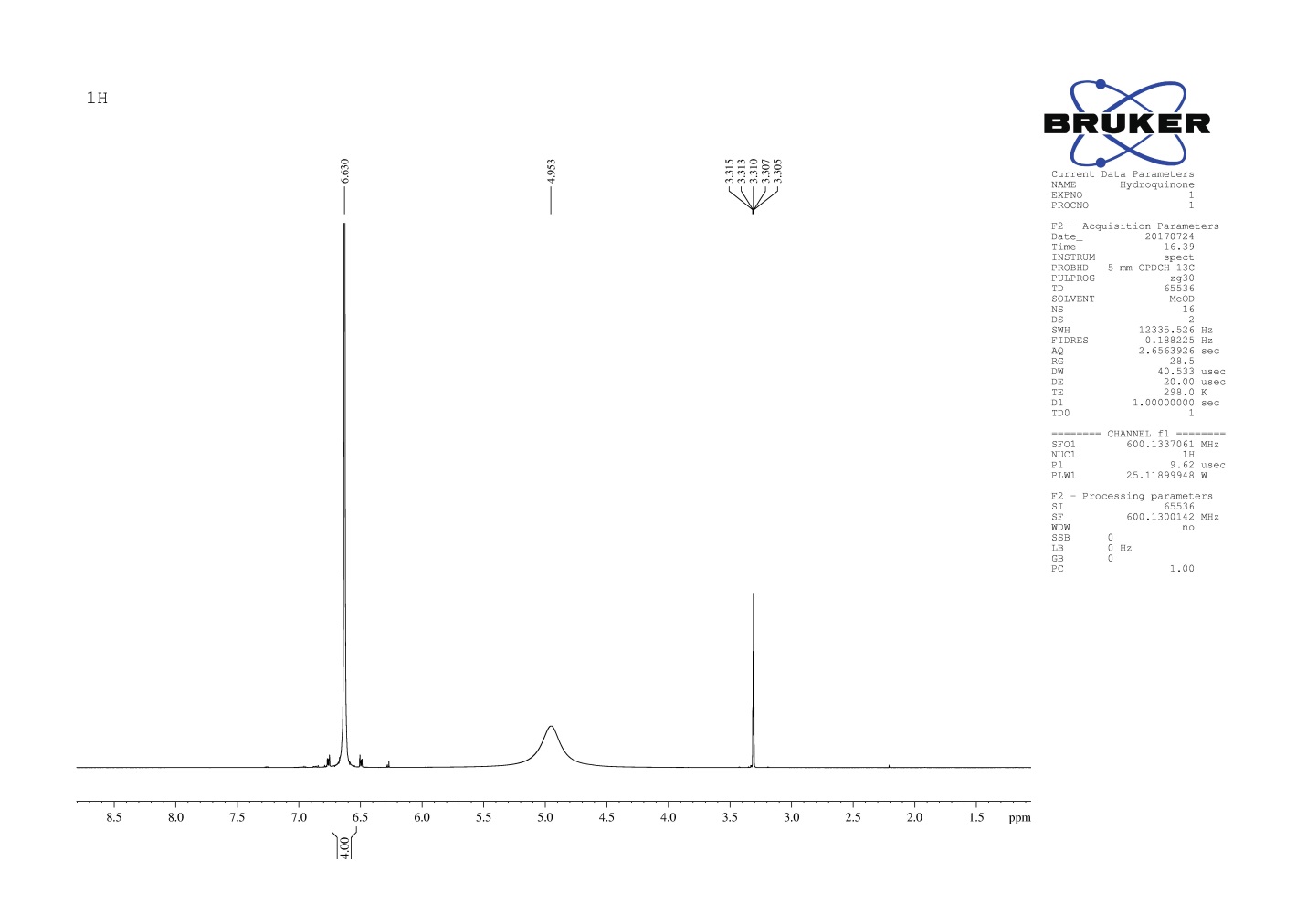
*

Figure 2. ^1^H-NMR spectrum of compound 1

*
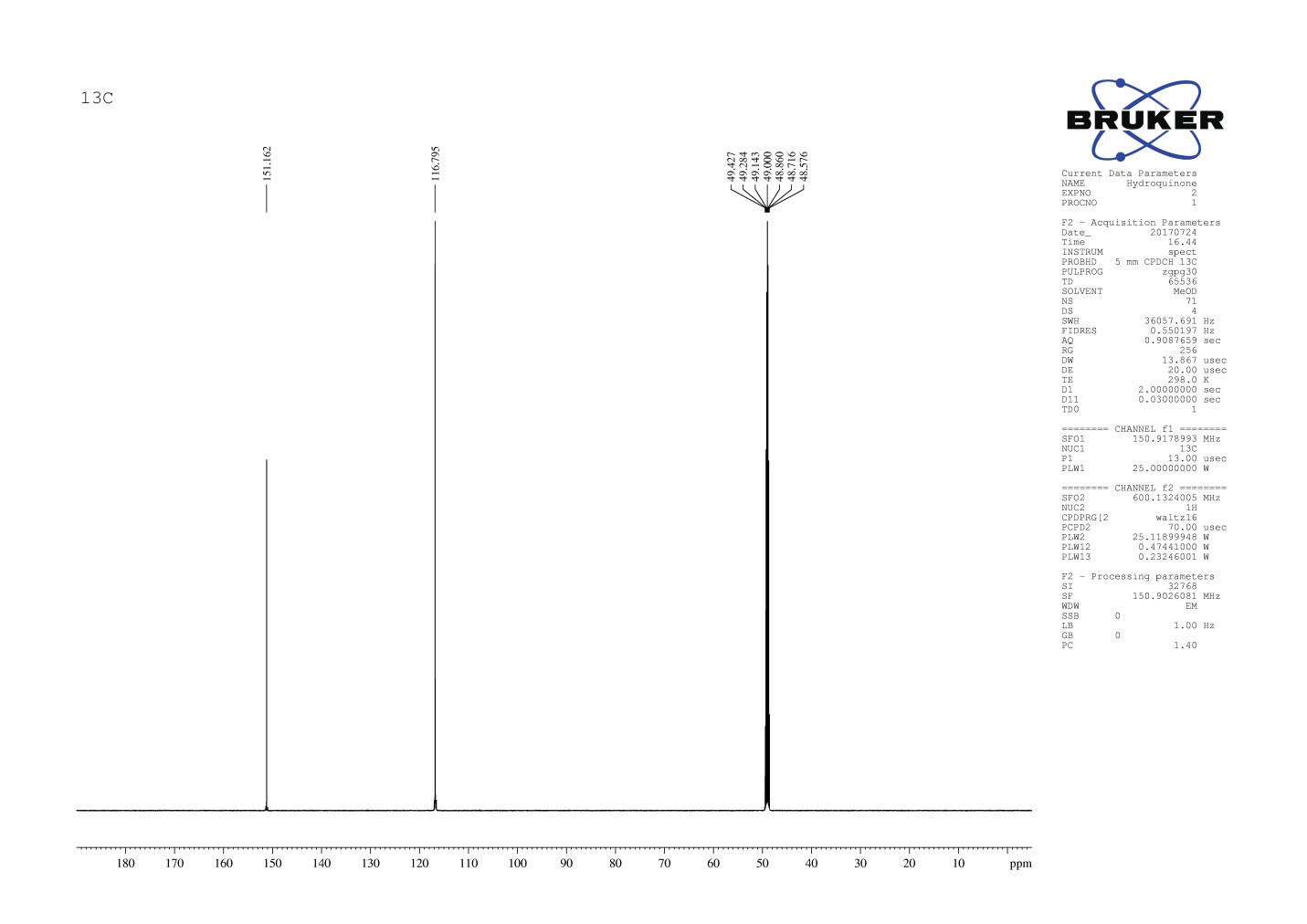
*

Figure 3. ^13^C-NMR spectrum of compound 1

|  |
| --- |
|  |

Figure 4: HRMS spectrum of compound 2

| Formula | Score | *m/z* (Calc) | Diff(ppm) | Ion Formula | *m/z* |
| --- | --- | --- | --- | --- | --- |
| C_10_H_10_O_4_ | 100 | 195.0652 | 1.4 | C_10_H_11_O_4_ | 195.0655 |
| C_10_H_10_O_4_ | 100 | 193.0506 | 2.5 | C_10_H_9_O_4_ | 193.0502 |
| 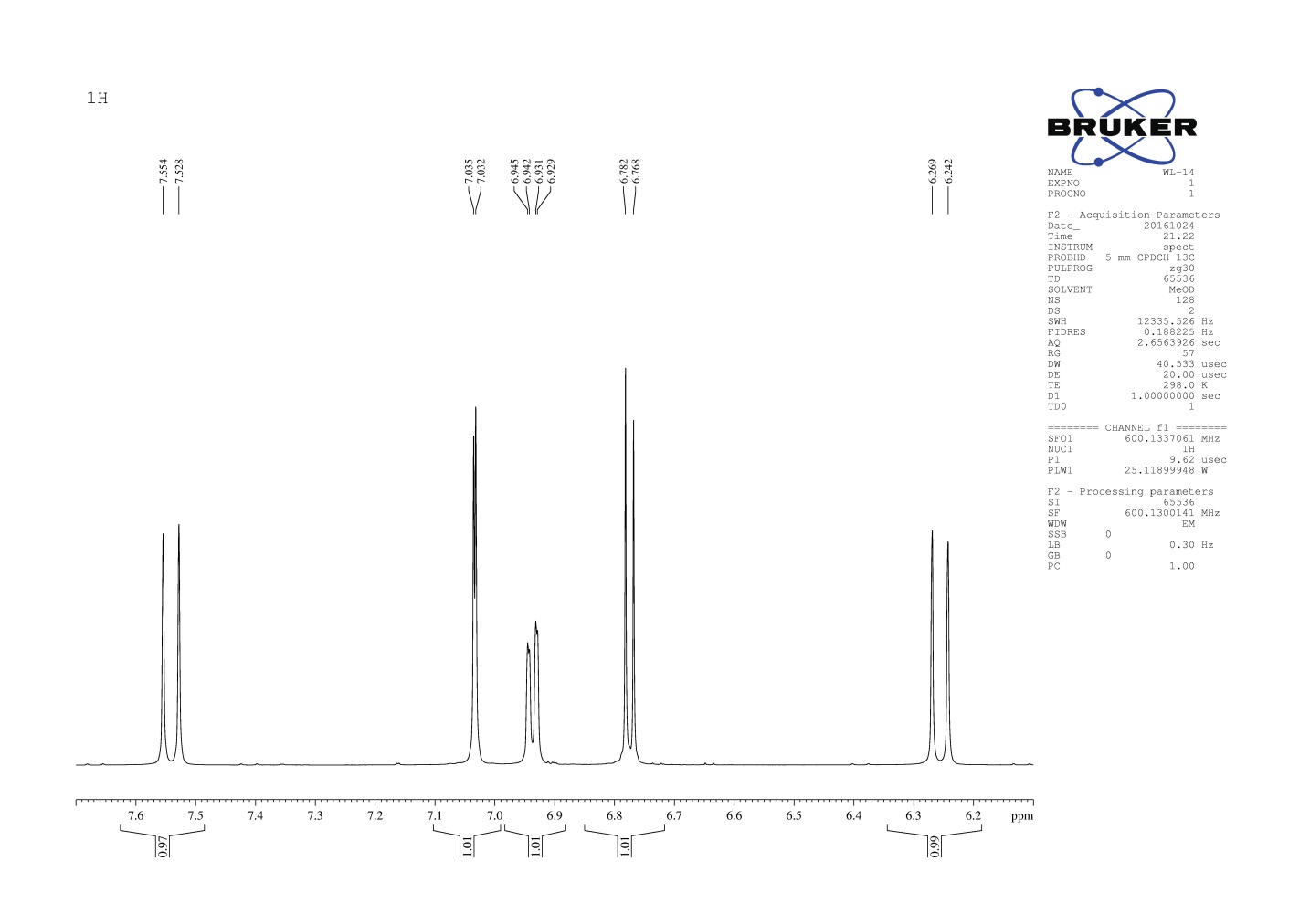 | | | | | |

Figure 5. ^1^H-NMR spectrum of compound


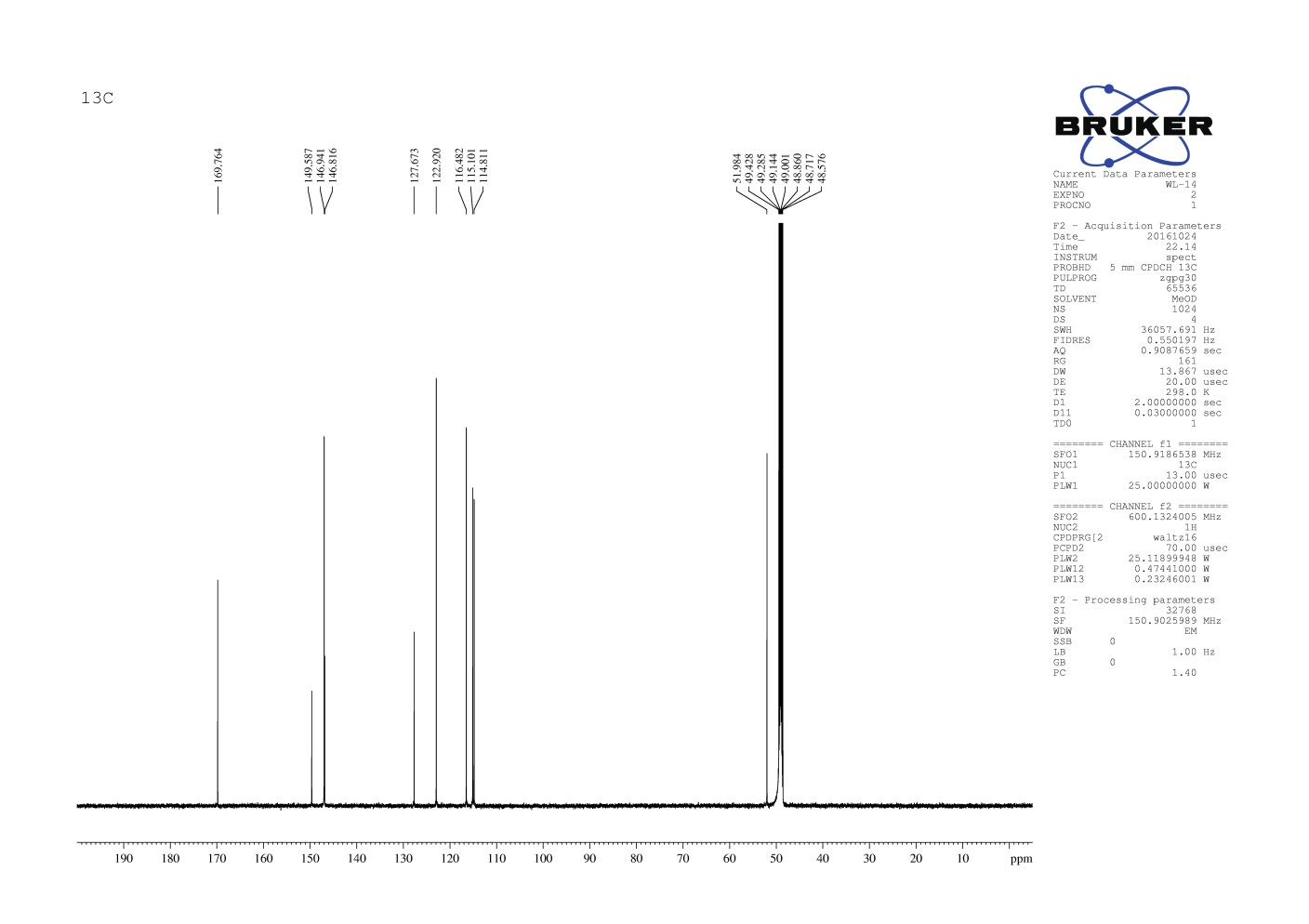
Figure 6. ^13^C-NMR spectrum of compound 2

**
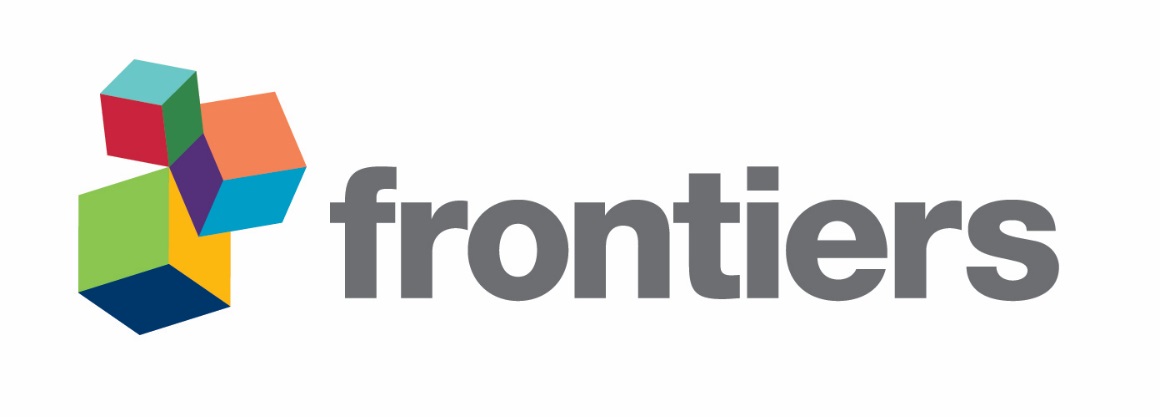
**
